# Endometrial gene expression profile of pregnant sows with extreme phenotypes for reproductive efficiency

**DOI:** 10.1038/srep14416

**Published:** 2015-10-05

**Authors:** S. Córdoba, I. Balcells, A. Castelló, C. Ovilo, J. L. Noguera, O. Timoneda, A. Sánchez

**Affiliations:** 1 Departament de Genètica Animal, Centre de Recerca en Agrigenòmica (CRAG), Universitat Autònoma de Barcelona (UAB), 08193 Bellaterra, Spain; 2 Departamento de Mejora Genética Animal, Instituto Nacional de Investigación y Tecnología Agraria y Alimentaria (SGIT-INIA), 28040 Madrid, Spain; 3 Genètica i Millora Animal, Institut de Recerca i Tecnologia Agroalimentàries (IRTA), 25198 Lleida, Spain

## Abstract

Prolificacy can directly impact porcine profitability, but large genetic variation and low heritability have been found regarding litter size among porcine breeds. To identify key differences in gene expression associated to swine reproductive efficiency, we performed a transcriptome analysis of sows’ endometrium from an Iberian x Meishan F_2_ population at day 30–32 of gestation, classified according to their estimated breeding value (EBV) as high (H, EBV > 0) and low (L, EBV < 0) prolificacy phenotypes. For each sample, mRNA and small RNA libraries were RNA-sequenced, identifying 141 genes and 10 miRNAs differentially expressed between H and L groups. We selected four miRNAs based on their role in reproduction, and five genes displaying the highest differences and a positive mapping into known reproductive QTLs for RT-qPCR validation on the whole extreme population. Significant differences were validated for genes: *PTGS2* (p = 0.03; H/L ratio = 3.50), *PTHLH* (p = 0.03; H/L ratio = 3.69), *MMP8* (p = 0.01; H/L ratio = 4.41) and *SCNN1G* (p = 0.04; H/L ratio = 3.42). Although selected miRNAs showed similar expression levels between H and L groups, significant correlation was found between the expression level of *ssc-miR-133a* (p < 0.01) and *ssc-miR-92a* (p < 0.01) and validated genes. These results provide a better understanding of the genetic architecture of prolificacy-related traits and embryo implantation failure in pigs.

Pig is economically one of the most important species. Reproductive traits such as fertility and prolificacy can directly impact porcine profitability, becoming one of the most relevant traits from a genetic and economic point of view. The annual production of a sow is determined to a large degree by its litter size in terms of total number of piglets born (TNB) and number of piglets born alive (NBA) per parity. Total number of piglets born and NBA are the most important reproductive traits used in swine breeding programmes^1^.

Although sow’s fertility depends directly on the ovulation rate (OR), litter size is not strongly determined by this factor, but by the capacity of maintaining viable embryos throughout gestation. Prenatal mortality could be a determinant factor for litter size in pigs^2^,^3^. The relevance and timing of embryonic and foetal losses during gestation have been reported in many studies, and it is estimated that about 25–45% of fertilized ova do not survive through gestation. Losses of embryos and foetuses occur at each stage of development and are primarily determined by the uterine capacity of the pregnant sows^4^. A large genetic variation has been found among porcine breeds regarding litter size, being the Chinese Meishan one of the most prolific pig breeds known^5^.

Improvements in litter size across the swine industry have occurred through different selection schemes such as phenotypic, family index, best linear unbiased prediction or hyper-prolific-based selection methods^2^. Being a complex trait regulated by a large number of genes, along with its low heritability, has made the selection of this character rather challenging for a number of years^6^. To date, main used strategies to detect those genes affecting litter size and its components have been: linkage analyses based on the identification of genomic regions linked with a phenotypic reproduction trait and candidate gene approaches, based on a priori knowledge of a gene having a high probability to play a relevant role in reproduction by their physiological role or location^7^.

Significant quantitative trait loci (QTL) associated with porcine reproductive traits have been identified in our study population and many others: SSC3, SSC8, SSC9, SSC10 and SSC15 for ovulation rates^8^,^9^,^10^,^11^, SSC7, SSC8, SSC12, SSC13, SSC14 and SSC17 for total number piglets born^6^,^12^,^13^, SSC4 and SSC13 for number of stillborn^10^,^14^ and SSC8 for uterine capacity and prenatal survival^13^,^15^. Although there are even more QTLs reported for litter size component traits, most of these results are inconsistent and true causal genes still remain scant due to the large disequilibrium linkage blocks present in the genome of livestock species^16^.

In recent years, the knowledge obtained by deciphering the pig genome and advances in molecular genetics, such as the transcriptomic analysis by RNA sequencing, have provided a powerful tool to better understand the genetic architecture of prolificacy-related traits. Recent years have seen a remarkable rise in porcine transcriptomic data. The use of microarrays and large-scale transcriptome analysis to identify differentially expressed genes in specific tissues, cell types or breeds has shed light on many aspects of porcine production traits^17^,^18^,^19^,^20^,^21^,^22^,^23^,^24^. Despite this, there have only been a few comparative studies on uterine function for prolific pigs and a low number of experiments regarding differences in endometrial gene expression between porcine breeds^25^,^26^,^27^.

In swine, during the oestrus cicle and throughout pregnancy many critical morphological and secretory changes take place in the uterus. These sets of physiological changes are clear evidence of the extremely complex interactions taking place between gene products and of remarkable transcriptomic reorganization. This highlights the importance of performing profiling experiments in porcine breeds with extreme prolificacy phenotypes, in order to better understand those gene interactions and the regulatory mechanisms affecting litter size in pigs.

An important mechanism of gene expression regulation is miRNAs. It is well known that miRNAs have key functions in many relevant biological processes, including cellular differentiation, proliferation, and apoptosis^28^. All these processes are involved in embryo formation, early development, and implantation. Although the exact role of miRNAs in normal embryo formation and endometrial preparation for pregnancy still remains unclear, they have been widely associated with mammalian development^29^. Moreover, Yu *et al.*, demonstrated that miRNA expression in mouse embryos was higher than in mature mouse tissues, confirming their role during embryo development^30^.

The goal of our study is, then, to define those genes and miRNAs that are differentially expressed in the uterine endometrium of pregnant sows with extreme prolificacy phenotypes in an Iberian x Meishan F_2_ population. These two porcine breeds differ significantly in their prolificacy levels, being the Meishan breed one of the most prolific porcine breeds, with an average of 14.3 piglets born alive per parity^31^, whereas the Iberian breed is considered a very low-prolificacy breed with an average of 7 piglets per parity^32^. This makes our study population highly suitable for further investigating the biological underpinnings that contribute to controlling litter size in pigs.

## Results

### Differential gene expression

Uterine receptivity to implantation is a process that can be very different, depending on the species, but always involves several changes in the expression of genes that are directly involved in pathways, such as progesterone and oestrogen biosynthesis, immune recognition, membrane permeability, angiogenesis and vasculogenesis, transport of nutrients and signalling for pregnancy recognition. Thus, changes in the expression level of those genes may influence uterine receptivity to implantation. Analysis of read counts revealed a total of 141 differentially expressed genes (DEG) between high- and low-prolificacy samples when a false discovery rate (FDR) corrected *q-value* of 0.05 was set as the threshold for significance (supplementary table S1). Expression differences between H and L groups ranged from 5.61 to −5.84 fold. A total of 55 transcripts showed an overexpression in the high-prolificacy group, with expression differences ranging from −1.45 to −5.84 fold, whereas 49 showed an overexpression in the low-prolificacy group, with expression changes ranging from 1.51 to 5.61 fold. Moreover, we identified 27 transcripts expressed uniquely in the L group (2 annotated genes and 25 unannotated transcripts) and 10 transcripts expressed uniquely in the H group, including 4 annotated genes and 6 unannotated transcripts (See supplementary table S2).

### Functional annotation and QTL mapping analysis

In order to establish whether differentially expressed genes found were involved in a relevant biological process for any stage of pregnancy establishment and development in the pig, we performed a gene ontology (GO) annotation and enrichment analysis. Obtained results revealed that the top over-represented functions were related with female pregnancy (*q-value* = 0.0001), maternal placenta development (*q-value* = 0.024) and decidualization (*q-value* = 0.024). All *p-values* were estimated through Chi square analysis and FDR corrected. An FDR-corrected *q-value* of 0.10 was set as the threshold for significant functional enrichment (See table 1). We also performed this enrichment analysis considering separately those genes overexpressed in either group. The DEG overexpressed in H prolificacy samples were clustered in seven enriched general biological processes, including mainly: positive regulation of cell proliferation (GO: 0008284; *q-value* =  3.67E-06) and response to hypoxia (GO: 0001666; *q-value* =  0.0002). Differentially expressed genes showing an overexpression in L prolificacy samples were clustered in 11 enriched general biological processes, including mainly: proteolysis/cell-cell signalling (GO: 0006508, GO: 0007267; *q-value* = 2.36E-06) and *in utero* embryonic development (GO: 0001701; *q-value* = 0.0001).

In order to focus on those genes that could be strongly associated with reproduction and have an impact on litter size variation, a chromosomal localization of DEGs within known QTL intervals was performed. We identified a total of 59 mapping into known reproductive QTLs. Among them, 25 were located within a QTL specifically related with litter size: total number of piglets born alive (NBA), total number of piglets born (TNB), total number of piglets stillborn (TSB), body weight at birth (BW), body weight at 10 weeks (WT), body weight at weaning (WWT), mummified pigs (MMUM) and/or ovulation rate (OVRATE). Results are shown in supplementary table S3.

### Candidate genes selection and expression levels validation: RT-qPCR

**Figure 1:**
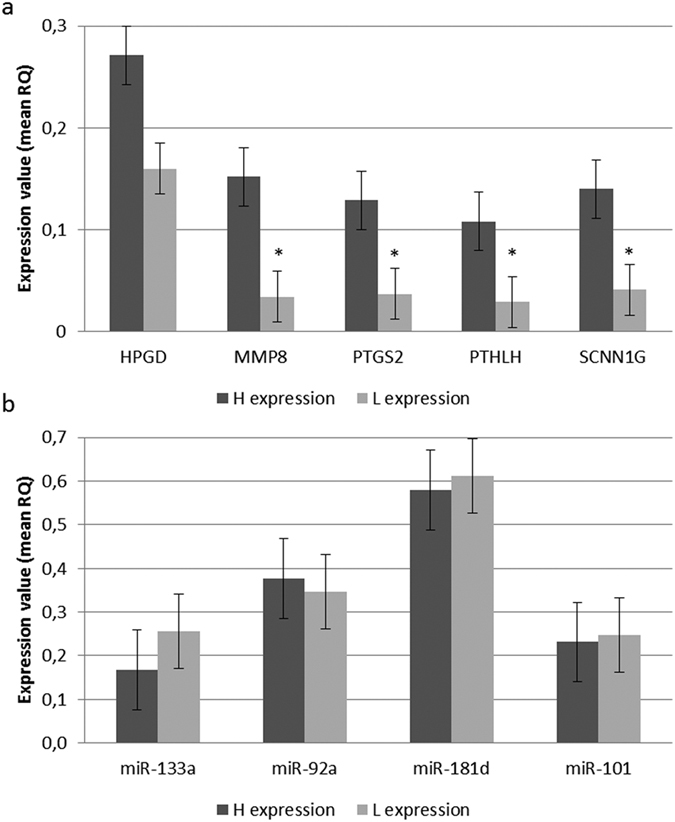
(**a**) RT-qPCR analysis results for gene expression. Expression values were calculated applying the −2^∆∆CT^ algorithm. Estimated relative quantities were normalized for the expression value of two uterus endogenous genes *B2MG* and *UBC* and calibrated to the sample with a higher expression. Significance was set at a *p-value* < 0.05 (*). (**b**) RT-qPCR analysis results for miRNA expression. Relative quantities were calculated using target-specific amplification efficiencies and normalized for the expression level of two uterus reference miRNAs: *has-miR-93* (M = 0.464; CV = 0.156 and *ssc-miR-103* (M = 0.464; CV = 0.166).

Among the 141 genes found differentially expressed in the RNAseq analysis (*q-value* < 0.05), we selected those displaying the most extreme differences between H and L groups (fold change ≥ 3) reducing the initial set to 28 genes. Based on the results obtained after the QTL mapping, we considered only those that have a positive mapping into known reproductive QTLs, reducing this number to 14 genes. Finally, considering the gene ontology (GO) annotation and enrichment analysis results and based on their known role in any relevant pathway related with reproduction, pregnancy or embryonic development, we chose 5 candidates: *HPGD*, *MMP8*, *PTGS2*, *PTHLH* and *SCNN1G* (See Table 2). Expression data obtained by RNA sequencing for these candidate genes was validated by RT-qPCR in 36 extreme individuals (H, n = 18; L, n = 18) of our F_2_ population. We confirmed significant differences in the expression level of four of these five genes between H and L samples with an H/L ratio > 3.5: *MMP8* (mean H = 0.174, mean L = 0.035; *p-value* = 0.011), *PTGS2* (mean H = 0.144, mean L = 0.038; *p-value* = 0.026), *PTHLH* (mean H = 0.126, mean L = 0.033; *p-value* = 0.034) and *SCNN1G* (mean H = 0.117, mean L = 0.031; *p-value* = 0.048). Results are shown in Fig. 1a. The observed ratios between the expression level of selected candidate genes were similar in our RNAseq and RT-qPCR analysis: *HPGD* (RNAseq FC = 1.85, RT-qPCR FC = 1.81), *PTGS2* (RNAseq FC = 4.06, RT-qPCR FC = 3.79), *PTHLH* (RNAseq FC = 4.32, RT-qPCR FC = 3.78) and *SCNN1G* (RNAseq FC = 3.65, RT-qPCR FC = 3.72). Only for the *MMP8* gene the observed ratios between both analysis were slightly different (RNAseq FC = 2.99, RT-qPCR FC = 4.92).

### Differential miRNA expression and *in silico* target prediction

The observed differences in the expression level of these genes between H and L prolificacy groups suggests that a different regulation mechanism may be occurring. We hypothesize that known gene regulators such as miRNAs could be responsible for this. Sequencing analysis revealed a total of 341 miRNAs being expressed in H and 329 in L prolificacy samples. Among all expressed microRNAs found in our endometrial samples, a total of 10 mature miRNAs were predicted as differentially expressed between H and L prolificacy phenotypes when considering a *p-value* < 0.05. However, we lost this significance when applying the same FDR correction significance criteria as used for DEG identification (Supplementary table S4).

To explore the possible regulatory role of these differentially expressed miRNAs, we predicted their potential target genes using TargetScan software. Five of these 10 differentially expressed miRNAs had as a putative mRNA target one of the DEGs found between the H and L groups (Supplementary table S5). The novel prediction tool from the mirDeep package allowed us to also identify 15 putative novel miRNAs in H samples and 12 in L samples, with an estimated probability of being a genuine miRNA precursor greater than 90% (Supplementary table S6).

### Candidate miRNAs selection and expression levels validation: RT-qPCR

Among the 10 miRNAs found differentially expressed in the RNAseq analysis (*q-value* < 0.05), we selected as candidates those that have been extensively reported in the literature as relevant in the regulation of reproduction-related genes in both pig and human: *ssc-miR-92a*, *ssc-miR-101*, *ssc-miR-133a* and *ssc-miR-181d* (See Table 3). We validated their expression levels by RT-qPCR in the same 36 F_2_ extreme individuals (H, n = 18; L, n = 18) used for gene expression validations (Table 3).

Obtained results revealed similar expression levels between both prolificacy groups for these four miRNAs (Fig. 1b). However, significant correlations were found between the expression level of prolificacy-related miRNAs *ssc-miR-92a* and *ssc-miR-133a* and validated DEG analysed by RT-qPCR (Table 4). Again, the observed fold changes were similar in both analysis: *ssc-miR-92a* (RNAseq FC = 1.26, RT-qPCR FC = 1.09), *ssc-miR-101* (RNAseq FC = 1.20, RT-qPCR FC = 0.94), *ssc-miR-181d-5p* (RNAseq FC = 1.16, RT-qPCR FC = 0.95). This confers consistency to our findings and led us to think that the observed differences in the expression levels between H and L groups represent the real biological background of our samples.

### Biological role of candidate genes: Interactions and upstream regulators

**Figure 2:**
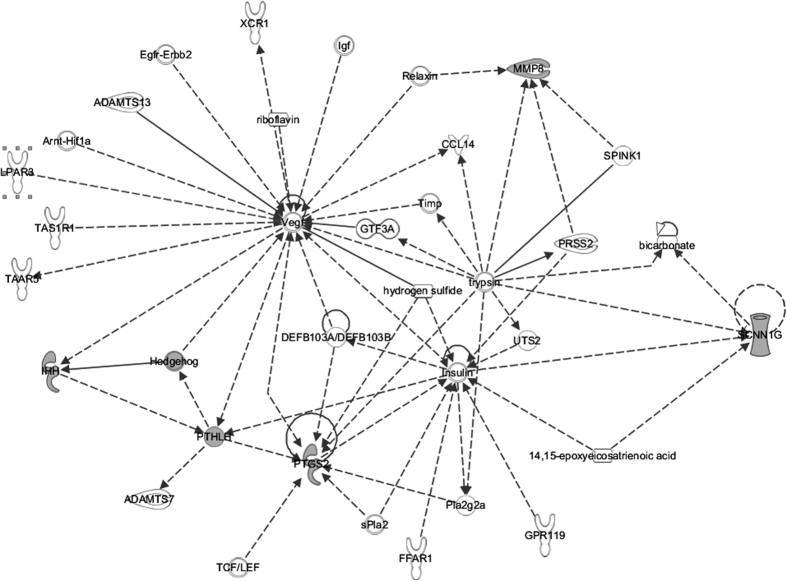
Ingenuity Pathway Analysis (IPA) Core Analysis-based network. Links of validated genes and other genes or molecules are represented with a continuous (direct interaction) or discontinuous line (indirect interaction).

To place the results in a biological context that allows us to better understand them, we performed an Ingenuity Pathway Analysis (IPA) to analyze the existing networks and potential molecular interactions between the validated candidate genes. Along pregnancy, hormones and other molecules secreted from the porcine conceptus act directly on the endometrium promoting its interaction with maternal uterus and placental development. We identified multiple links and interactions between our validated candidate genes and some molecular components. In the predicted network generated by IPA algorithm (Fig. 2), we observed that the expression of our four validated candidates could be modulated mainly by three molecules: trypsin (for genes *MMP8*, *PTGS2* and *SCNN1G*), insulin (for gene *SCNN1G*) and the vascular endothelial growth factor (*Vegf*) which acts on *PTHLH* gene.

After performing the analysis of the putative common upstream regulators we identified that the common regulators to all four genes are the cytokines Interleukin 1 beta (*ILK-1β*, *p-value* = 0.000007) and the tumor necrosis factor ligand (*TNF*, *p-value* = 0.00008). Results are shown in Table 5.

### Discussion

In this study, we investigated the whole transcriptome profile of the swine endometrial epithelium in an Iberian x Meishan F_2_ population using RNA sequencing (RNA-seq), with the aim to identify key differences in gene expression associated to swine reproductive efficiency. Understanding the complexity of the key mechanisms for successful reproduction in humans and animals has been challenging. Even though a few studies have addressed this goal, this study represents one of the first descriptions of the mechanisms that affect embryonic survival in the pig, providing the knowledge to enhance fertility and reproductive health in this species.

The main limitation of increasing litter size in pigs is prenatal mortality. Two critical stages are early and mid-gestation, responsible for around 20–30% (days 10–30 of gestation) and 10–15% (days 50–70 of gestation) of embryonic loss respectively^2^. Recent evidences have indicated that the prenatal loss in pigs results mainly from the decreased placental efficiency and uterine capacity^33^,^34^.

Uterine receptivity to implantation is a process that can be very different, depending on the species, but always involves several changes in the expression of genes that are directly involved in pathways, such as progesterone and oestrogen biosynthesis, immune recognition, membrane permeability, angiogenesis and vasculogenesis, transport of nutrients and signalling for pregnancy recognition^35^,^36^. Thus, changes in the expression level of those genes may influence uterine receptivity to implantation.

In this study we have identified 141 differentially expressed genes between high and low prolificacy samples. Functional enrichment analysis suggested that most of these genes are directly involved in the above-mentioned biological processes, which are highly relevant for pregnancy and some specific stages of embryonic development in swine. We have focused our validations on a first set of genes that are up-regulated in our high-prolificacy samples. Some of those genes are also located inside the confidence intervals of previously described reproduction QTLs: ovulation rate, gestation length, number of piglets born alive and embryo’s birth weight. Considering these, we proceeded to validate their expression by real time RT-qPCR. As predicted in the RNAseq analysis, four of these genes were differentially expressed in our endometrial samples, being overexpressed in those with a high-prolificacy phenotype.

Several DEGs found in our samples have been extensively discussed by many authors before^37^,^38^,^39^,^40^,^41^,^42^, and their involvement in the establishment of pregnancy and in the physiological, molecular and structural changes that take place in the uterine tissue to promote embryo implantation have been demonstrated in pigs and other mammals . Their involvement in many stages of embryonic development postulate them as key factors for deciphering the mechanisms involved in the regulation of litter size in our study population.

Prostaglandins (PGs) produced by the uterus play an important role in regulation of the oestrous cycle and during early pregnancy in pigs and many other species^43^. In the porcine endometrium, luteoprotective *PGE2* and luteolytic *PGF2α* are the main PGs produced and pregnancy establishment depends directly in a proper ratio between the synthesis of both. An inhibition of PG synthesis results in pregnancy failure^44^. One of the validated genes found differentially expressed among our samples is the prostaglandin endoperoxide synthase (*PTGS*; also known as prostaglandin G/H synthase or cyclooxygenase *COX2*). The *PTGS2* gene has been widely discussed over the years and its key function to ensure reproductive success has been widely demonstrated through several previous studies. It constitutes a rate-limiting enzyme in the production of PGs as it catalyzes the conversion of arachidonic acid to *PGH2*, which is a common substrate for various prostaglandins. Its conserved role in implantation in various species, including humans, has previously been discussed^45^,^46^. Thus, considering that the production of prostaglandins directly contributes to the successful establishment of pregnancy, and that uterine receptivity to implantation is progesterone-dependent, a lack in the expression of this gene will directly affect the appropriate conceptus attachment. It has been observed that the expression of *PTGS1* and *PTGS2* is substantially increased during implantation. We speculate that the underexpression of this gene in our low-prolificacy samples may contribute to embryonic deaths due to deficiencies in progesterone synthesis. This uterine receptivity via expression of *PTGS2* gene is a process that has been demonstrated to be directly regulated by another key gene also found DE in our samples: *KLF5*. This gene belongs to the Kruppel-like factors (KLFs) family. This is a zinc finger-containing transcription factor, which is known to regulate several cellular processes, including development, differentiation, proliferation, and apoptosis^47^. At the beginning of the attachment reaction, the first cell type to interact with the blastocyst trophectoderm is the uterine luminal epithelium. *KLF5* function is critical to make this uterine luminal epithelium conducive to blastocyst implantation and growth. In its absence, trophectoderm development is defective, resulting in developmental arrest at the blastocyst stage^48^. These results suggest that *KLF5* is a key regulator of embryo pre-implantation^49^. Thus, the fact that this gene is overexpressed in our high-prolificacy samples strengthens our idea of the important effect it may have on prolificacy levels and litter size control.

As mentioned before, successful establishment of pregnancy also depends on many structural changes that take place in the uterine tissue. Species with invasive implantation require a cell-to-cell communication through connexin proteins. Although porcine implantation is superficial, some authors have reported that endometrial cell-to-cell interaction may also be necessary for limiting trophoblast invasiveness or to develop specific channels that allow this superficial implantation^50^. And it is at this stage where the validated gene *MMP8* plays a key role. Proteins such as matrix metalloproteinase (MMP) are a family of enzymes (with more than 20 members identified) that use zinc-dependent catalysis to break down the components of the extracellular matrix (ECM)^39^,^51^,^52^. We hypothesize that the observed significant overexpression of this gene in our high-prolificacy samples may indicate a more efficient tissue reorganization to support the growing foetus.

Another relevant structural gene found differentially expressed in our extreme F_2_ population is the Forkhead transcription factor *FOXA2*. *FOXA* transcription factors are a subfamily of Forkhead transcription factors that has been found to play an important role in early development, organogenesis, metabolism and homeostasis^53^. Low-prolificacy samples show a decreased expression of this gene compared to those with high prolificacy, supporting our idea that an underexpression of this gene could be leading to defects in early development, affecting stages such as gastrulation or, later on, in embryo morphogenesis.

Many other genes found differentially expressed in this study are closely related with critical stages in embryo development at implantation level or later in the survival of the embryo itself. This has provided us with a powerful list of candidates that require further validations in order to prove their direct involvement in the control of litter size in swine. Because of the usefulness of the pig as a biomedical model and the parallelism in the function of these genes in humans, this study also provides a powerful tool to understand which genes are key in the process of embryo survival in mammals.

We also wanted to explore the regulatory mechanisms that do mediate this differential expression in our study population. To do so, we have also analysed the miRNA expression profile in both extreme phenotypic groups.

We predicted a differential expression of 10 mature miRNAs between our H and L prolificacy samples. Some of these differentially expressed miRNAs have been demonstrated to be directly involved in the regulation of reproductive-related genes in pig and other mammals^54^,^55^,^56^,^57^. After this preliminary bioinformatic screening we proceeded to the experimental validation of the expression level of 4 of these 10 miRNAs, considering their role in reproductive-related pathways: *ssc-miR-92a*, *ssc-miR-101*, *ssc-miR-133a* and *ssc-miR-181d*.

In concordance with RNAseq predictions, *ssc-miR-101*, *ssc-miR-133a* and *ssc-miR-181d* were overexpressed in L samples while *ssc-miR-92a* was overexpressed in H samples. *MiR-92*, belongs to the *miR-17* ~ *92* cluster, demonstrated in recent reports to regulate cardiac development, endothelial cell proliferation and angiogenesis, which are relevant processes for embryogenesis and pregnancy itself^58^. Loss and gain of function experiments showed that miR-92a inhibited angiogenesis *in vitro* and *in vivo*^59^ and that deletion of *miR-92a* is sufficient to induce a developmental skeletal defect^55^. Thus, the observed overexpression of this miRNA in our H samples could be explained by its positive effect on several key processes for pregnancy and embryo development.

Real-time RT-qPCR analysis revealed similar expression levels of these miRNAs in both groups (FC < 1.5). However, it has been demonstrated that even very small changes in microRNA expression levels (FC 1.5 to 2.5) could have a direct impact on their target genes and some authors have observed these small differences when performing miRNA differential expression studies related to reproductive processes^60^,^61^. We hypothesize that this could be caused by an insufficient sequencing depth in our libraries, because despite these similar miRNA expression levels observed between both phenotypes, a significant correlation was found between the expression levels of validated genes *PTHLH*, *MMP8*, *PTGS2* and *SCNN1G*, and both *ssc-miR-133a* and *ssc-miR-92a*. Therefore, the finding of this significant correlation leads us to think that the observed differences, despite being low, may be biologically significant. Many years ago, Calin *et al.* suggested that the capability of miRNAs to regulate multiple targets within the same pathway could amplify their biological effects^62^.

Besides miRNAs, upstream regulators such as transcription factors (TFs), growth factors (GFs) and many other molecules may play a critical role as *drivers* or master regulators of gene expression. Investigating their involvement in a particular gene network or pathway can provide better clues on the underlying regulatory mechanisms that do mediate the observed differences in the expression of key genes in a particular biological context.

In this study we have explored the regulatory role that some candidate miRNAs exert in the expression of key reproductive-related genes and the possible effect that this has on litter size control. In addition, we have established which interactions exist between our validated candidate genes and other known regulatory molecules. There are two cytokines particularly capable of acting on the expression of these four genes which are the *ILK-1β* and the *TNF*.

In reproductive biology, the role of these cytokines has been implicated in ovulation, menstruation, and embryo implantation, and pathological processes such as preterm delivery, and endometriosis^63^,^64^. The interleukin 1 is a pro-inflammatory cytokine with multiple functions in a range of tissues^65^. All components of the IL-1 system have been examined in the human endometrium and have been implicated as an important mediator of embryo implantation^66^,^67^. Simón C. and collaborators, demonstrated in mice, that IL-1 receptor antagonist given before implantation significantly reduces the number of implanted embryos, indicating a role for IL-1 in embryo implantation^64^.

The TNF is a pro-inflammatory cytokine that plays an important role in modulating the acute phase reaction. It was first discovered in amnion and placenta^68^, but many studies have demonstrated the presence of this cytokine and its receptors in the diverse human reproductive tissues^69^. The TNF has been implicated in ovulation, corpus luteum formation and luteolysis, and it has been related to many endometrial and gestational diseases such as amniotic infections, recurrent spontaneous abortions, preeclampsia, preterm labour or endometriosis^70^,^71^,^72^. Although these cytokines may be acting on the expression of our validated candidate genes, we haven’t seen them differentially expressed between H and L groups.

It is clear, that there is a complex network of interacting genes regulating litter size in pigs. However, this work has led to the identification of several potential candidate genes associated with critical steps involved in embryonic survival during the sow’s gestation. Our results also describe the possible regulatory mechanisms that could be responsible of the differences in the expression level of key genes related with litter size control in pigs.

## Materials and Methods

### Animal material and sample collection

Animals used in this study come from an F_2_ population resulting from the cross of 3 Iberian males from the Guadyerbas line (Dehesón del Encinar, Toledo, Spain) with 18 Meishan females (Domaine du Magneraud, INRA, France). Once the F_1_ generation was obtained, 8 boars and 97 sows were mated to obtain a 255 F_2_ progeny at the Nova Genètica S.A. experimental farm (Lleida, Spain).

During four consecutive parities, main parameters based on the sows’ reproductive efficiency were recorded: number of piglets born alive (NBA) and total number of piglets born (TNB) means. At day 30–32 of their fifth gestation, when litter size has reached the maximum^73^, sows were slaughtered and the number of *corpora lutea* (CL or OR) and number of foetuses (NF) attached to the uterus were also recorded. At slaughter, endometrial samples from the apical uterus of F_2_ sows were collected and subsequently snap-frozen in liquid nitrogen. Preservation and storage was made at −80 °C until usage. All animal procedures were carried out according to the European animal experimentation ethics law and approved by the institutional animal ethics committee of Institut de Recerca i Tecnologia Agroalimentàries (IRTA).

### Phenotypic records and samples selection

F_2_ sows were ranked by their estimated breeding value (EBV), which was calculated by using best linear unbiased predictors (BLUP) according to the reproductive traits described above: NBA and TNB means, OR and NF. Based on this ranking, individuals were divided into two groups: high (H; EBV > 0) and low (L; EBV < 0) prolificacy. Among the whole F_2_ progeny (n = 255), individuals displaying the most extreme EBVs were selected to be used in this study (n = 36). All phenotypic records are shown in Table 6.

### RNA isolation and quality assessment

Total RNA was extracted from sows’ endometrial samples using TRIzol® reagent (Invitrogen, Carlsbad, USA), following the manufacturer’s instructions. The RNA integrity was assessed using an Eukaryote Total RNA Nano 6000 Labchip on an Agilent 2100 Bioanalyzer (Agilent Technologies, Palo Alto, USA) and quantified using a NanoDrop ND-1000 spectrophotometer (NanoDrop Technologies, Wilmington, USA). Only those RNA samples with an RNA integrity number (RIN) ≥ 7 were used in subsequent experiments.

### Ion Torrent PGM libraries preparation and RNA sequencing

Ion Torrent adapter-ligated libraries were prepared from extracted total RNA according to the Ion Total RNA-seq Kit v2 protocol (Life Technologies – Part #4476286 Rev. B) following the manufacturer’s instructions.

#### mRNA libraries preparation

Samples corresponding to animals displaying very extreme EBVs and very high RNA quality (RIN≥ 8) were used to prepare mRNA libraries (H, n = 3; L, n = 3). We constructed sequencing libraries starting from 500 ng of total RNA. PolyA RNA fraction was purified from total RNA samples using the Dynabeads^®^ mRNA DIRECT Micro Kit (Life Technologies – Part #1148804 Rev. A) following the manufacturer’s instructions. Each sample was subjected to Ion semiconductor sequencing using a 318 chip on an Ion-Torrent PGM sequencer.

#### Small RNA libraries preparation

Small RNA sequencing was also performed using 318 chips on an Ion-Torrent PGM sequencer. In this case, we used stored GS FLX 454 microRNA sequencing libraries that we had previously used in our research^61^, which included the same extreme samples used in the mRNA libraries protocol (H, n = 7; L, n = 5). To adapt these performed libraries to the Ion semiconductor sequencing technology protocol, it was necessary to remove the 454 specific adaptors and to add the Ion Torrent A and P1 specific ligators. After doing so, each miRNA library was re-sequenced.

### Bioinformatics and statistical analysis

Approximately 5 million short single-end reads (≈200 bp) were obtained for each library and sample and were subsequently assembled into a non-redundant set of 30,585 gene transcripts (3,024,658,544 bp) from the available *Sus scrofa* genome alignment version 10.2 (available at http://www.ncbi.nlm.nih.gov/assembly/GCF_000003025.5/#/def). In average, 75% of the reads were successfully mapped to the *Sus scrofa* genome.

#### Quality control for single-end raw reads

Raw reads formatted as fastq files were processed using FastQC 0.10.1 (freely available at http://www.bioinformatics.babraham.ac.uk/projects/fastqc/). Considered low quality reads by applying FastQC defaults, were removed and all downstream analyses were performed only on those reads meeting the quality criteria. Ion Torrent A and P1 adaptors were removed using Cutadap 1.4 (freely available at http://code.google.com/p/cutadapt/).

#### Reads mapping, alignment and annotation

Obtained sequence reads from mRNA libraries were mapped with Tophat (v1.4.0) to the latest porcine genome sequence assembly (Sscrofa10.2, August 2011). Transcript isoforms were assembled using Cufflinks 2.1.1 and combined with gene annotations extracted from Ensembl (ftp://ftp.ensembl.org/pub/release-75/gtf/sus_scrofa). The criteria used to filter out unique sequence reads was: minimum length fraction of 0.9; minimum similarity fraction of 0.8 and a maximum number of 2 mismatches.

Sequence reads from small RNA libraries were analysed following the Perl scripts contained in the miRDeep 2.0 package^74^ (freely available at http://www.mdc-berlin.de/rajewsky/miRDeep). Briefly, reads were first collapsed to ensure that each sequence only occurs once. Collapsed reads were then mapped to predefined miRNA precursor sequences from the miRBase v.20 contained in the porcine genome sequence assembly (Sscrofa 10.2, August 2011). Finally, unmapped reads served as input sequences for the novel miRNAs prediction algorithm.

### Differential gene expression, functional annotation and QTL mapping analysis

Analysis of differential gene expression across high and low-prolificacy groups was performed using Cuffdiff 2.0.2 which is included in the Cufflinks package (available at http://cufflinks.cbcb.umd.edu/manual.html). For small RNA libraries, differentially expressed miRNA genes were detected by using the DEseq R package 1.8.3^75^. A Benjamini-Hochberg FDR corrected *p-value* of 0.05 was set as the threshold for significant differential expression in both cases.

Babelomics 4.3.0 (http://babelomics.bioinfo.cipf.es) was used to functionally annotate DEG. The pig functional annotation database is not as complete as human, therefore, we converted the pig gene IDs (Ensembl *Sus scrofa* 10.2) into human gene IDs using Ensembl BioMart tool (http://www.ensembl.org/biomart/martview/). Then the homologous human Ensembl IDs were submitted to the Babelomics database for functional annotation. *P-values* to estimate over-represented GO terms were obtained through Chi square analysis. An FDR-corrected *p-value* of 0.10 was set as the threshold for significance.

All differentially expressed genes found were mapped against the latest release (Aug 25, 2014) of the Pig Quantitative Trait Locus Database^76^. Those DEGs displaying a significant functional annotation related to reproduction processes and/or a positive mapping into known reproductive QTLs were selected as a first set of candidates for quantitative real-time PCR validations.

### Expression level validation by reverse transcription quantitative real-time PCR (RT-qPCR)

Five candidate genes and four candidate miRNAs displaying significant differences in their expression level between H and L samples were validated by RT-qPCR. The same samples selected for RNA-seq were used in these validations, but in order to obtain a broader view of the expression level of these genes in our population, the sample size was expanded using other extreme F_2_ samples (H, n = 18; L, n = 18).

#### Reverse transcription (RT): cDNA synthesis

Extracted total RNA was quantified using an ND 1000 Nanodrop® Spectrophotometer (Thermo Scientific, Wilmington, USA). The RNA quality and integrity were determined using an Eukaryote Total RNA Nano 6000 Labchip on an Agilent 2100 Bioanalyzer (Agilent Technologies, Palo Alto, USA).

Synthesis of cDNA for gene expression validation was performed using the High Capacity cDNA Reverse Transcription kit (Applied Biosystems) from 1 μg of total RNA in 20 μl reaction. The synthesis of cDNA for miRNA expression validation was performed using extracted total RNA as described by Balcells *et al.*^77^ Briefly, 600 ng of total RNA in a final volume of 20 μL including 10x poly (A) polymerase buffer, 0.1 mM of ATP, 0.1 mM of each dNTP, 1 μM of RT-primer, 200 U of M-MuLV Reverse Transcriptase (New England Biolabs, USA) and 2 U of poly (A) polymerase (New England Biolabs, USA) was incubated at 42 °C for 1 hour and 95 °C for 5 minutes for enzyme inactivation. The used RT-primer sequence was 5′-CAGGTCCAGTTTTTTTTTTTTTTTVN, where V is A, C and G and N is A, C, G, and T. Minus reverse transcription (RT) and minus poly A) polymerase controls were performed.

#### Real-time RT-qPCR reaction

##### DE genes expression validation

Quantitative PCR reactions were performed in triplicate in 20 μL final volume including 10 μL SYBR® Select Master Mix (Life Technologies – Thermo Fisher Scientific, Massachusetts, USA), 300 nM of each primer and 5 μL of a 1:200 dilution of the cDNA. A 1:5 relative standard curve generated from a pool of equal amounts of cDNA from all samples was included in each qPCR assay to estimate qPCR efficiency. Reactions were incubated in a 96-well plate at 95 °C for 10 min, followed by 40 cycles of 95 °C for 15 sec and 60 °C for 1 min on a 7900 HT Real-Time PCR System using 7900HT SDS v2.4 software (Applied Biosystems, USA). DNA primers for each gene were designed using Primer Express® software v2.0 (Applied Biosystems, USA) following manufacturer’s instructions (Table 7). Melting curve analysis was included in each qPCR to detect unspecific amplifications. Expression values were calculated with qbasePLUS software (Biogazelle) applying the −2^∆∆Ct^ algorithm, after verifying that the assumptions of the method were met^78^. Estimated relative quantities were calibrated to the sample with a higher expression and normalized for the expression value of two uterus endogenous genes: *B2MG*^79^ and *UBC*^80^. Reference genes stability was also assessed with qBasePLUS software considering a GeNorm M value < 0.5 and a coefficient of variation (CV) < 0.2. Significance was set at a *p-value* < 0.05.

#### DE miRNAs expression validation and putative targets prediction

Quantitative PCR reactions were performed as described above but using a different concentration of primers according to each miRNA. DNA primers were designed following the methodology suggested by Balcells *et al.* (Table 8). Relative standard curves were included in each qPCR assay to estimate target-specific amplification efficiencies. Expression values were calculated with qbasePLUS software using these amplification efficiencies. Relative quantities were normalized for the expression value of two uterus reference miRNAs: *has-miR-93* and *ssc-miR-103*^81^ and calibrated to the sample with a higher expression. Reference miRNAs stability was determined considering a GeNorm M value < 0.5 and a coefficient of variation (CV) < 0.2. Significance was set at a *p-value* < 0.05.

Biological putative targets prediction was performed using TargetScan 6.2 online software. Targets were considered true positives if conserved 8mer and 7mer sites match the seed region of each miRNA.

### Analysis of candidate genes interactions and upstream regulators

The four validated genes (*MMP8*, *PTHLH*, *PTGS2* and *SCNN1G*) were submitted to Ingenuity Pathway Analysis (IPA 4.0, Ingenuity Systems Inc., www.ingenuity.com) for mapping to canonical pathways and identifying upstream regulators. As the Ingenuity Knowledge Base relies on ortholog information for only human, mouse, and rat, we submitted to IPA the correspondent human Ensembl IDs of our candidate genes. We ran the Core Analysis function designating a set of criteria: genes and endogenous chemicals, direct and indirect interactions, maximum molecules per network (35) and networks per analysis (25), humans as the selected specie, all tissues and primary cells. The resulting networks were scored based on the fold change provided by Cuffdiff as log_2_ (fold change) for each gene. The obtained *p-values* correspond to the Fisher’s exact test, with the null hypothesis that the molecules within the networks are connected based on chance.

## Additional Information

**How to cite this article**: Córdoba, S. *et al.* Endometrial gene expression profile of pregnant sows with extreme phenotypes for reproductive efficiency. *Sci. Rep.* **5**, 14416; doi: 10.1038/srep14416 (2015).

## Supplementary Material

Supplementary Information

## Figures and Tables

**Table 1:** Functional enrichment analysis showing the top significantly-over-represented GO terms in which identified DEG are involved.

**GO term**	**Biological process**	**Log OR**^a^	***p-value***	***q-value***^b^	**DEG involved**
GO:0007565	Female pregnancy	2.892	0.00000	0.0001	8
GO:0001893	Maternal placenta development	4.035	0.00004	0.0243	3
GO:0046697	Decidualization	4.218	0.00002	0.0243	3
GO:0048545	Response to steroid hormone	2.100	0.00004	0.0243	7
GO:0000038	Long-chain fatty acid metabolism	3.812	0.00007	0.0307	3
GO:0006694	Steroid biosynthetic process	2.267	0.00025	0.0722	5
GO:0009888	Tissue development	1.284	0.00030	0.0722	12
GO:0001503	Ossification	1.993	0.00026	0.0722	6
GO:0042127	Regulation of cell proliferation	1.264	0.00035	0.0722	12
GO:0060348	Bone development	1.957	0.00032	0.0722	6
GO:0009725	Response to hormone	1.605	0.00036	0.0722	8
GO:0001501	Skeletal system development	1.578	0.00043	0.0785	8
GO:0043129	Surfactant homeostasis	4.314	0.00056	0.0942	2
GO:0007398	Ectoderm development	1.813	0.00066	0.0969	6
GO:0051216	Cartilage development	2.396	0.00066	0.0969	4

^a^Odds ratio logarithmic transformation.

^b^Benjamini-Hochberg FDR-corrected *p-value.*

**Table 2:** Results summary for the selected candidate genes.

**Gene**	**Function**	**References**	**QTL**	**Enriched Biological Process**	RNA-seq analysis^a^		Log2FC	*q-value*	RT-qPCR analysis^b^		FC	***q-value***
					**High (RPKM)**	**Low (RPKM)**			**High (RQ)**	**Low (RQ)**		
*HPGD*	Biosynthesis of prostaglandins (PTG)	Atli MO *et al.* 2010	–	Female pregnancy (GO:0007565)	102.14	28.40	1.85	0.032	0.271	0.160	1.69	0.118
		Palliser HK *et al.* 2014										
		Kowalewski MP *et al.* 2014										
*MMP8*	Collagen metabolism and preeclampsia	Mousa AA *et al.* 2012	NNIP	Embryo development (GO:0009790)	68.49	8.64	2.99	0.008	0.152	0.034	4.44	0.013
	Remodeling of the cervical and fetal membrane ECM	Wang H *et al.* 2004										
*PTGS2*	Converts arachidonic acid to PGH2	Waclawik A. *et al.* 2011	GEST	Maternal placenta development (GO:0001893)	109.91	6.57	4.06	0.008	0.129	0.037	3.50	0.027
		Blitek A. t al. 2006										
	Rate-limiting enzymes in PG synthesis	Blitek A. *et al.* 2006										
		Sales KJ *et al.* 2003										
	Essential to reproduction	Murakami M. *et al.* 2004										
		Lim H *et al.* 1997										
		Langenbach R *et al.* 1999										
		Silver RM *et al.* 1995										
*PTHLH*	Nipple development during pregnancy	Martínez-Giner M *et al.* 2011	NSB	Lactation	286.09	14.36	4.32	0.008	0.108	0.029	3.69	0.027
	Preimplantation	Guo L *et al.* 2012										
	Fetoplacental development	Thota CS *et al.* 2005										
	Embryonic mammary development	Hiremath M *et al.* 2013										
*SCNN1G*	Preeclaampsia	Marino G *et al.* 2013	OVRATE	Response to hypoxia (GO:0001666)	33.29	2.65	3.65	0.008	0.140	0.041	3.42	0.048
			BW	Sodium ion transport (GO:0006814)								

^a^In the RNAseq analysis, expression values are shown as RPKM values (Reads per Kilobase of exon model per Million mapped reads) and mean difference between groups as the log_2_ transformed fold change (Log_2_FC).

^b^In the RT-qPCR analysis, expression values are shown as mean relative quantities (RQ) and mean difference between groups is represented as the fold change (FC).

**Table 3:** Results summary for the validated candidate miRNAs.

**miRNA**	**Function**	**References**	**DEG predicted as target**	**RNAseq analysis**^a^				**RT-qPCR analysis**^b^			
				**High (RPKM)**	**Low (RPKM)**	**Log2 FC**	***q-value***	**High (RQ)**	**Low (RQ)**	**FC**	***q-value***
*ssc-miR-92a*	Angiosenesis	Bellera N.*et al.*, 2014	*HPGD*	51,874.13	21,710.41	−1.26	0.032	0.376	0.347	1.09	0.515
	Embryo implantation	Su L.*et al.*, 2014									
	Placentation	Su L.*et al.*, 2014									
	trophoblast differentiation	Kumar P.*et al.*, 2013									
*ssc-miR-101*	Ginecological tumors	Torres A.*et al.*, 2010	*HTRA3, ATP1B1, PTGS2, JUNB*	430.21	187,19	−1.20	0.034	0.231	0.247	0.94	0.829
	Embryo implantation	Chakrabarty A.*et al.*, 2007									
	Endometriosis	Teague E.*et al.*, 2010									
*ssc-miR-133a*	Uterine tumors	Torres A.*et al.*, 2010	*ENPEP*	533.76	1,777.17	1.74	0.050	0.168	0.255	0.66	0.290
	Skeletal muscle development	Lee J.*et al.*, 2013									
*ssc-miR-181d*	Hypoxia	Shen G.*et al.*, 2013	*MMP8, MME*	55.51	124.35	1.16	0.046	0.580	0.611	0.95	0.698
	Embryo implantation	Su L.*et al.*, 2014									
	Placentation	Su L.*et al.*, 2014									
	Endometrial stromal decidualization	Estella C.*et al.*, 2012									

^a^In the RNAseq analysis, expression values are shown as RPKM values (Reads per Kilobase of exon model per Million mapped reads) and mean difference between groups as the log_2_ transformed fold change (Log_2_FC).

^b^In the RT-qPCR analysis, expression values are shown as mean relative quantities (RQ) and mean difference between groups is represented as the fold change (FC).

**Table 4:** Pearson’s correlations between miRNA expression values obtained by RT-qPCR and validated target genes expression.

		***MMP8***	***PTGS2***	***PTHLH***	***SCNN1G***
*ssc-miR-133a*	Pearson’s correl.	−0.575	−0.537	−0.533	−0.516
	*p-value*	0.0003*	0.0007*	0.0008*	0.0013*
	N	36	36	36	36
*ssc-miR-181d*	Pearson’s correl.	−0.140	−0.139	−0.088	−0.137
	*p-value*	0.4159	0.4199	0.6113	0.4240
	N	36	36	36	36
*ssc-miR-101*	Pearson’s correl.	−0.059	−0.045	−0.069	−0.123
	*p-value*	0.7380	0.7889	0.6925	0.4824
	N	35	35	35	35
*ssc-miR-92a*	Pearson’s correl.	0.630	0.574	0.615	0.551
	*p-value*	0.00004*	0.0002*	0.00007*	0.0005*
	N	36	36	36	36

Significance was set at a *p-value* < 0.05. ^(*)^Correlation is significant at the 0.01 level (bilateral).

**Table 5:** Network associations of upstream regulators and validated candidate genes predicted by Ingenuity Pathway Analysis (IPA).

**Upstream Regulator**	**Molecule Type**	***p-value***	**Target molecules**
Dexamethasone	Chemical drug	0.000001	*MMP8,PTGS2,PTHLH,SCNN1G*
*IL1B*	Cytokine	0.000007	*MMP8,PTGS2,PTHLH,SCNN1G*
*TNF*	Cytokine	0.000079	*MMP8,PTGS2,PTHLH,SCNN1G*
Lipopolysaccharide	Chemical drug	0.000097	*MMP8,PTGS2,PTHLH,SCNN1G*

The Core Analysis calculates the predicted upstream regulators based on the FC direction (up-regulated or down-regulated) observed among known downstream targets.

**Table 6:** Phenotypic records of the F_2_ Iberian × Meishan sows used in this study.

**Prolificacy level**	**Animal**	**NBA**^a^	**TNB**^a^	**OR**^b^	**NF**^b^	**EBV**
HIGH	A1 (791)	12.00	10.00	13.00	10	1.73
	A2 (787)^c^,^d^	11.75	13.00	16.00	16	1.68
	A3 (169)	12.25	11.00	14.00	11	1.68
	A4 (332)^c^,^d^	12.75	13.33	16.00	14	1.55
	A5 (373)^c^,^d^	11.25	11.00	20.00	17	1.50
	A6 (878)^d^	12.00	10.50	14.00	7	1.42
	A7 (425)	11.00	11.00	0.00	13	1.34
	A8 (767)	9.40	10.50	17.00	14	1.31
	A9 (20)	11.00	10.00	20.00	14	1.22
	A10 (127)	11.00	11.67	17.00	13	1.21
	A11 (365)	10.50	10.00	16.00	9	1.17
	A12 (389)^d^	10.25	10.50	19.00	16	1.09
	A13 (597)	10.00	9.50	20.00	11	0.92
	A14 (151)	10.75	12.00	20.00	13	0.89
	A15 (874)^d^	10.25	10.00	11.00	8	0.82
	A16 (271)	10.50	9.67	15.00	14	0.81
	A17 (30)	10.75	10.67	19.00	13	0.80
	A18 (485)	11.00	12.50	16.00	16	0.77
Average (HIGH)		**11.02**	**10.94**	**15.72**	**12.72**	**1.22**
LOW	A19 (350)^c^,^d^	4.50	3.00	15.00	6	−2.48
	A20 (309)	5.00	4.33	16.00	8	−2.42
	A21 (360)^c^,^d^	5.00	5.33	18.00	1	−2.33
	A22 (260)	4.75	5.00	17.00	10	−2.31
	A23 (173)	5.00	6.67	15.00	10	−2.30
	A24 (861)^c^,^d^	5.50	5.00	24.00	9	−2.04
	A25 (409)	4.75	5.67	18.00	11	−1.94
	A26 (918)	7.00	8.50	16.00	13	−1.46
	A27 (779)	6.25	5.50	23.00	10	−1.45
	A28 (915)	4.75	4.00	18.00	8	−1.21
	A29 (443)	5.25	6.50	16.00	5	−1.13
	A30 (702)	6.00	7.50	13.00	11	−1.06
	A31 (322)^d^	4.75	5.00	16.00	14	−0.95
	A32 (204)	5.00	3.67	14.00	15	−0.95
	A33 (486)^d^	5.25	3.50	24.00	5	−0.91
	A34 (499)	6.75	6.50	13.00	11	−0.59
	A35 (895)	7.25	8.50	13.00	10	−0.46
	A36 (846)	6.75	5.00	22.00	14	−0.45
Average (LOW)		**5.53**	**5.51**	**17.28**	**9.50**	−**1.47**

^a^NBA (number of piglets born alive) and TNB (total number of piglets born) trait entries correspond to the average for four consecutive parities.

^b^OR (number of *corpora lutea*) and NF (number of foetuses) recorded at slaughter on the fifth gestation.

^c^Extreme samples used for mRNA libraries preparation and sequencing.

^d^Extreme samples used for microRNA libraries sequencing.

**Table 7:** Primers used for the genes RT-qPCR validation design.

**Gene**	**Forward Primer**	**Reverse Primer**	**Type**	**Conc.**
*B2MG*	ACCTTCTGGTCCACACTGAGTTC	GGTCTCGATCCCACTTAACTATCTTG	Endogenous	300 nM
*HPGD*	CAGGCACAACTTAGAGATACATTTAGG	TCCAGCATTATTGACCAAAATGTC	Target gene	300 nM
*MMP8*	GGACCAAAACCTCCAAAAATTACA	TGAGACAGCCCCAAGGAATG	Target gene	300 nM
*PTGS2*	ACGAGCAGGCTGATACTGATAGG	GTGGTAGCCACTCAGGTGTTGTAC	Target gene	300 nM
*PTHLH*	GCCGCCGACTCAAAAGAG	CGCCGTAAATCTTGGATGGA	Target gene	300 nM
*SCNN1G*	GCTGCCTACTCCCTGCAGATC	TACTGAGCGCACCCACATTTC	Target gene	300 nM
*UBC*	GCATTGTTGGCGGTTTCG	AGACGCTGTGAAGCCAATCA	Endogenous	300 nM

**Table 8:** Primers used for the miRNAs RT-qPCR validation design.

**miRNA**	**miRNA Sequence**	**Forward Primer**	**Reverse Primer**	**Conc.**
*hsa-miR-93*	CAAAGTGCTGTTCGTGCAGGTAG	GCAAAGTGCTGTTCGTG	TCCAGTTTTTTTTTTTTTTTCTACCT	200 nM
*ssc-miR-92a-2*	TATTGCACTTGTCCCGGCCTGT	AGGTGTGTATAAAGTATTGCACTTGTCC	CAGGTCCAGTTTTTTTTTTTTTTTACAG	250 nM
*ssc-miR-101-1*	TACAGTACTGTGATAACTGAA	GCTGTATATCTGAAAGGTACAGTACTGTGAT	GGTCCAGTTTTTTTTTTTTTTTCAGTT	250 nM
*ssc-miR-103*	AGCAGCATTGTACAGGGCTATGA	AGAGCAGCATTGTACAGG	GGTCCAGTTTTTTTTTTTTTTTCATAG	250 nM
*ssc-miR-133a-1*	TTGGTCCCCTTCAACCAGCTG	GAATGGATTTGGTCCCCTTCA	CAGTTTTTTTTTTTTTTTCAGCTGGT	250 nM
*ssc-miR-181d-5p*	AACATTCAACGCTGTCGGTGAGTT	CACAATCAACATTCATTGTTGTCG	TCCAGTTTTTTTTTTTTTTTAACCCAC	250 nM
