# Supplementary material for: Endometrial gene expression profile of pregnant sows with extreme phenotypes for reproductive efficiency
Source: Sci Rep. 2015 Oct 5;5:14416. doi: 10.1038/srep14416 (PMC5155628; doi:10.1038/srep14416)
Supplement: Supplementary Information [file srep14416-s1.pdf]

Endometrial gene expression profile of pregnant sows with extreme phenotypes for reproductive efficiency

S. Córdoba[1,*], I. Balcells[1], A. Castelló[1], C. Ovilo[2], JL. Noguera[3], O. Timoneda[1], A. Sánchez[1]

[1] Departament de Genètica Animal, Centre de Recerca en Agrigenòmica (CRAG), Universitat Autònoma de Barcelona (UAB), 08193 Bellaterra, Spain

[2] Departamento de Mejora Genética Animal, Instituto Nacional de Investigación y Tecnología Agraria y Alimentaria (SGIT-INIA), 28040 Madrid, Spain

[3] Genètica i Millora Animal, Institut de Recerca i Tecnologia Agroalimentàries (IRTA), 25198 Lleida, Spain

*sarai.cordoba@cragenomica.es

Table S1. Differential expressed genes found in mRNA libraries between both extreme phenotypes. All expression values are shown as RPKM values (Reads per Kilobase of exon model per Million mapped reads – Mortazavi et al., 2008). Mean difference between both groups is represented as the $\log_2$ transformed fold change (Log_2FC).

Gene name	**Locus**	**High RPKM**	**Low RPKM**	**Log_2 FC**	**p-value**	**q-value[a]**	**Up regulation**
Un-annotated	2:120052627-120052759	6159,840	1190,200	-2.372	0.0001	0.0129	High
ADM	2:52576214-52578540	165,926	35,818	-2.212	0.0004	0.0319	High
ANXA8	14:95804060-95825709	134,914	10,747	-3.650	0.0001	0.0078	High
ATP1B1	4:89529320-89550560	213,255	18,941	-3.493	0.0001	0.0078	High
BF	7:27771731-27777630	17,525	4,625	-1.922	0.0003	0.0282	High
CES1	6:27276810-27380644	88,817	10,330	-3.104	0.0001	0.0078	High
CST6	2:5395319-5396971	904,334	236,311	-1.936	0.0002	0.0173	High
CXCL16	12:54264905-54269592	185,155	36,204	-2.354	0.0002	0.0173	High
DF	2:77485871-77487847	79,457	21,607	-1.879	0.0002	0.0173	High
DPCD	14:122343533-122381270	81,013	18,670	-2.117	0.0006	0.0370	High
EGLN3	7:70667588-70695692	28,945	3,381	-3.098	0.0001	0.0078	High
ENSSSCG00000004703	1:142682190-142688725	162,847	29,120	-2.483	0.0001	0.0129	High
ENSSSCG00000010533	14:119116727-119128192	103,147	1,800	-5.840	0.0005	0.0360	High
ENSSSCG00000012427	X:69067109-69087469	1318,870	245,174	-2.427	0.0003	0.0282	High
ENSSSCG00000013976	2:55147384-55155430	175,303	5,964	-4.877	0.0001	0.0078	High
ENSSSCG00000026236	13:170261824-170278122	142,493	38,629	-1.883	0.0001	0.0129	High
ENSSSCG00000027404	1:3101176-3126386	59,013	12,192	-2.275	0.0008	0.0454	High
ENSSSCG00000027784	16:51499064-51507622	35,405	8,666	-2.030	0.0003	0.0262	High
ENSSSCG00000028525	2:44078430-44082946	135,308	17,341	-2.964	0.0004	0.0319	High
ENSSSCG00000028923	10:309238-337906	34,982	2,141	-4.030	0.0001	0.0078	High
EPHA1	18:7081287-7098452	22,152	6,946	-1.673	0.0003	0.0282	High
EYA2	17:54887015-55078442	30,370	7,571	-2.004	0.0002	0.0215	High
FXYD3	6:40111306-40118025	211,400	22,672	-3.221	0.0001	0.0078	High
GPR110	7:48466208-48513535	38,595	8,630	-2.161	0.0001	0.0078	High
HDC	1:135147966-135166937	20,811	1,531	-3.765	0.0003	0.0282	High
HMGCR	2:85967320-85990095	56,566	12,800	-2.144	0.0001	0.0129	High
HPGD	14:16908629-16921262	102,135	28,404	-1.846	0.0004	0.0319	High
HSD17B7	4:95571647-95594215	75,112	21,034	-1.836	0.0002	0.0173	High
KLF5	11:49867087-49870148	210,133	53,936	-1.962	0.0005	0.0360	High
LCN2	1:302600678-302605199	80,798	16,342	-2.306	0.0001	0.0078	High
MAPK4	1:110082309-110155111	44,643	7,117	-2.649	0.0001	0.0078	High
MMP8	9:37330737-37343663	68,489	8,639	-2.987	0.0001	0.0078	High
MSLN	3:41448661-41452015	40,196	4,376	-3.199	0.0002	0.0215	High
MST1R	13:35590300-35603228	28,392	7,735	-1.876	0.0002	0.0173	High
MTMR11	4:108596807-108604443	38,519	11,268	-1.773	0.0002	0.0173	High
MUC1	4:103409754-103413841	148,217	22,219	-2.738	0.0003	0.0282	High
MUC4	13:143786442-143842402	44,827	10,970	-2.031	0.0004	0.0319	High

NOP56	17:37441072-37451309	219,218	36,853	-2.573	0.0005	0.0352	High
OVOL1	2:5630670-5642984	22,256	4,696	-2.245	0.0001	0.0078	High
PARP3	13:37272322-37280891	21,833	8,022	-1.445	0.0007	0.0433	High
PLA2G4A	9:140460879-140623439	17,008	4,535	-1.907	0.0003	0.0282	High
PSAT1	1:257809814-257835728	38,254	10,211	-1.906	0.0007	0.0414	High
PTGS2	9:140251515-140260362	109,913	6,574	-4.063	0.0001	0.0078	High
PTHLH	5:49160138-49172386	286,097	14,359	-4.316	0.0001	0.0078	High
RAB25	4:102703921-102713495	87,765	20,834	-2.075	0.0001	0.0078	High
SCNN1G	3:23444853-23480156	33,295	2,647	-3.653	0.0001	0.0078	High
SDCBP2	17:38419446-38437273	49,494	6,487	-2.932	0.0001	0.0078	High
SFN	6:77758723-77760027	66,169	11,685	-2.501	0.0001	0.0078	High
SGPP2	15:137861023-137905123	11,590	1,686	-2.781	0.0001	0.0078	High
SLC52A3	17:39142052-39160998	13,635	2,891	-2.238	0.0007	0.0433	High
SMOC1	7:99860647-99938608	42,954	6,162	-2.801	0.0001	0.0078	High
STAP2	2:74955494-74964488	24,900	4,142	-2.588	0.0003	0.0282	High
TMEM139	18:7211799-7214244	65,159	4,274	-3.930	0.0001	0.0078	High
TMEM79	4:102462253-102467485	28,115	5,337	-2.397	0.0006	0.0370	High
VEGF,VEGFA	7:44224281-44475316	992,547	60,231	-4.043	0.0001	0.0078	High
Un-annotated	2:76981417-76982104	34,069	112,967	1.729	0.0005	0.0360	Low
Un-annotated	3:10903133-10908516	12,831	46,010	1.842	0.0002	0.0173	Low
Un-annotated	3:19921984-19925331	20,020	83,411	2.059	0.0003	0.0262	Low
APOA1	9:49288614-49290784	18,310	88,047	2.266	0.0001	0.0078	Low
CEBPD	4:87368560-87610320	25,189	89,790	1.834	0.0008	0.0454	Low
CFL2	7:69919876-69924154	42,519	146,918	1.789	0.0005	0.0360	Low
CHRAC1	4:2425324-2427426	38,895	119,120	1.615	0.0004	0.0308	Low
CLEC3B	13:31097019-31106889	47,058	176,399	1.906	0.0002	0.0215	Low
CYP17A1	14:123773104-123779533	4,570	52,641	3.526	0.0001	0.0078	Low
DCLK2	8:83617784-83721850	14,485	51,711	1.836	0.0007	0.0433	Low
DPT	4:89969323-90001121	17,523	51,942	1.568	0.0005	0.0360	Low
ECHDC1	1:39547701-39593566	6,554	29,560	2.173	0.0001	0.0129	Low
ENDOD1	9:30923140-30952504	7,300	29,739	2.026	0.0001	0.0078	Low
ENPEP	8:119968857-120061196	6,394	49,590	2.955	0.0001	0.0078	Low
ENSSSCG00000000921	5:97776302-97793667	5,701	17,837	1.646	0.0006	0.0370	Low
ENSSSCG00000004572	1:121176825-121178352	2,404	46,442	4.272	0.0008	0.0454	Low
ENSSSCG00000004573	1:121348386-121353049	23,755	205,948	3.116	0.0001	0.0078	Low
ENSSSCG00000008627	3:133981946-134005017	12,734	51,289	2.010	0.0004	0.0319	Low
ENSSSCG00000010464	14:112652686-112657270	13,535	45,323	1.744	0.0001	0.0129	Low
ENSSSCG00000013152	2:12110927-12113060	7,494	235,166	4.972	0.0001	0.0129	Low
ENSSSCG00000017492	12:22869394-22876022	31,662	89,836	1.505	0.0009	0.0499	Low
ENSSSCG00000025083	1:108567669-108607804	37,358	232,497	2.638	0.0004	0.0319	Low
ENSSSCG00000026285	6:90674121-90703000	10,746	41,930	1.964	0.0004	0.0319	Low
ENSSSCG00000029421	1:268964059-268973843	7,241	41,506	2.519	0.0001	0.0129	Low
EPC1	10:47431312-47541566	19,907	210,948	3.406	0.0001	0.0078	Low
FAM174B	7:92211760-92247931	7,952	26,642	1.744	0.0004	0.0308	Low
FOXA2	17:34053459-34056624	9,494	37,100	1.966	0.0001	0.0129	Low
GPER	3:828570-833135	3,927	24,583	2.646	0.0001	0.0078	Low

HBE1	9:5650627-5652665	20,916	1024,720	5.614	0.0001	0.0078	Low
HTRA3	8:4456175-4482055	19,965	77,677	1.960	0.0004	0.0319	Low
IHH	15:134122694-134129391	5,928	28,747	2.278	0.0002	0.0215	Low
JUNB	2:66505143-66507024	25,216	79,818	1.662	0.0004	0.0308	Low
KLF2	1:279056680-279061593	7,126	22,844	1.681	0.0007	0.0433	Low
MGP	5:61054165-61058328	157,765	502,327	1.671	0.0007	0.0414	Low
MME	13:103030829-103128447	26,332	85,476	1.699	0.0009	0.0499	Low
MMP23B	6:58231350-58233984	7,099	55,587	2.969	0.0001	0.0078	Low
MYEOV2	15:153946103-153950517	27,781	86,956	1.646	0.0007	0.0414	Low
NEXN	6:125500472-125693958	11,924	60,290	2.338	0.0001	0.0078	Low
PDK4	9:82625076-82638263	22,654	72,263	1.673	0.0005	0.0360	Low
PI15	4:66776707-66802989	9,080	30,478	1.747	0.0001	0.0078	Low
PION	9:113183480-113208758	2,916	14,502	2.314	0.0009	0.0499	Low
RGS5	4:94999825-95057119	23,315	131,159	2.492	0.0002	0.0173	Low
ROR2	14:3602842-3643321	6,403	18,823	1.556	0.0005	0.0360	Low
SAL1	1:284447109-284451960	4,702	37,998	3.015	0.0006	0.0391	Low
SH3BGR	13:213286139-213348206	14,455	58,620	2.020	0.0001	0.0078	Low
SLC24A4	7:120438866-120615704	7,877	34,496	2.131	0.0006	0.0370	Low
SST	13:134620965-134622407	12,591	155,456	3.626	0.0001	0.0129	Low
TM9SF2	11:75703401-75712182	36,818	144,519	1.973	0.0006	0.0370	Low
WFDC1	6:4754997-4789463	7,011	19,908	1.506	0.0006	0.0391	Low

Table S2. Differentially expressed genes found uniquely expressed in one of the prolificacy groups in mRNA libraries. All expression values are shown as RPKM values (Reads per Kilobase of exon model per Million mapped reads). Shown *q-values* are Benjamini-Hochberg false discovery rate (FDR) corrected *p-values*. Mean difference between both groups is represented as the log_2 transformed fold change (Log_2FC).

Gene name	**Locus**	**High RPKM**	**Low RPKM**	***p-value***	***q-value***	**Expression**
un-annotated	1:305102825-305126687	0,000	22,438	0.0001	0.0078	low group
un-annotated	10:63437508-63437903	0,000	15,437	0.0003	0.0282	low group
un-annotated	11:86787469-86788850	0,000	21,723	0.0004	0.0308	low group
un-annotated	11:49276974-49322148	0,000	38,516	0.0005	0.0360	low group
un-annotated	11:306177-306599	0,000	11,421	0.0006	0.0370	low group
un-annotated	12:6776624-6776820	0,000	101,847	0.0005	0.0360	low group
un-annotated	12:13704609-13705014	0,000	24,158	0.0001	0.0078	low group
un-annotated	13:3656448-3695027	0,000	57,550	0.0001	0.0078	low group
un-annotated	13:34286207-34286740	0,000	30,630	0.0006	0.0391	low group
un-annotated	13:191013924-191014235	0,000	28,185	0.0002	0.0215	low group
un-annotated	15:117232153-117235744	0,000	17,595	0.0001	0.0078	low group
un-annotated	15:122380337-122380596	0,000	77,571	0.0001	0.0078	low group
un-annotated	16:77861325-77861582	0,000	36,153	0.0008	0.0480	low group
un-annotated	2:74436079-74436163	0,000	1755,170	0.0004	0.0319	low group
un-annotated	2:146140303-146140525	0,000	141,576	0.0001	0.0078	low group
un-annotated	3:133864573-133867381	0,000	60,757	0.0001	0.0078	low group
un-annotated	3:144233946-144234106	0,000	190,345	0.0006	0.0370	low group
un-annotated	4:95670907-95689264	0,000	50,452	0.0006	0.0370	low group
un-annotated	4:53167877-53167899	0,000	386984	0.0001	0.0078	low group
un-annotated	6:54625398-54625615	0,000	61,993	0.0005	0.0352	low group
un-annotated	6:71273302-71273742	0,000	14,237	0.0004	0.0308	low group

un-annotated	7:82944504-82947401	0,000	67,349	0.0001	0.0078	low group
un-annotated	7:69929899-69930246	0,000	26,751	0.0001	0.0078	low group
un-annotated	9:133471303-133471700	0,000	14,237	0.0004	0.0319	low group
un-annotated	X:37199874-37200061	0,000	117,755	0.0006	0.0391	low group
CCDC23	4:117723360-117723558	0,000	95,426	0.0003	0.0282	low group
ENSSSCG00000021428	2:137744797-137747670	0,000	34,076	0.0001	0.0078	low group

Gene name	**Locus**	**High RPKM**	**Low RPKM**	***p-value***	***q-value***	**Expression**
un-annotated	1:146326137-146332733	19,218	0,000	0.0001	0.0078	high group
un-annotated	1:153401809-153402129	30,745	0,000	0.0001	0.0078	high group
un-annotated	13:116302834-116303158	70,331	0,000	0.0001	0.0078	high group
un-annotated	15:140262241-140262584	22,064	0,000	0.0004	0.0308	high group
un-annotated	3:100325999-100326309	50,622	0,000	0.0001	0.0078	high group
un-annotated	6:51851923-51854793	14,580	0,000	0.0001	0.0078	high group
ENSSSCG00000009447	11:34047436-34047881	14,081	0,000	0.0001	0.0129	high group
KLK1	6:51469506-51475249	13,053	0,000	0.0001	0.0078	high group
MYL4	12:16799290-16813119	17,438	0,000	0.0001	0.0078	high group
NMU	8:58581166-58601463	152,849	0,000	0.0001	0.0078	high group

Table S3. QTL mapping results for those DEGs located within at least one QTL closely related with litter size. Pocine breeds correspond to: Largewhite (LW), ME (Meishan), Landrance (LD), Duroc (DU), Pietrain (PT), Yorkshire (YS) and French Landrace (French LD).

DEG	Locus	QTL	QTL ID	QTL name	QTL coordinates (bp)	Breed
CES1	6:27276810-27380644	**TNB**	QTL:24281	Total number born	Chr.6:8144130-116389124	LW, ME
DCLK2	8:83617784-83721850	TNUM	QTL:4253	Teat number	Chr.8:38873367-90653103	
		**TNB**	QTL:24282	Total number born	Chr.8:3470575-143577862	LW, ME
		**OVRATE**	QTL:492	Corpus luteum number	Chr.8:52718097-133922781	
ENSSSCG00000000921	5:97776302-97793667	**NSB**	QTL:18128	Number of stillborn	Chr.5:231749-108789684	LW, LD
FXYD3	6:40111306-40118025	**TNB**	QTL:24281	Total number born	Chr.6:8144130-116389124	LW, ME
GPER	3:828570-833135	**OVRATE**	QTL:4249	Corpus luteum number	Chr.3:14776389-23924094	
		TNUM	QTL:5224	Teat number	Chr.3:1802851-131434519	LW, ME
		**DRIPL**	QTL:5692	Drip loss	Chr.3:14776389-62780348	DU, PT
		**BW**	QTL:5694	Body weight (birth)	Chr.3:14776389-62780348	DU, PT
KLK1	6:51469506-51475249	**TNB**	QTL:24281	Total number born	Chr.6:8144130-116389124	LW, ME
MMP23B	6:58231350-58233984	**TNB**	QTL:24281	Total number born	Chr.6:8144130-116389124	LW, ME
NEXN	6:125500472-125693958	**TNB**	QTL:10620	Total number born	Chr.6:74531339-129740986	ME, YS
NMU	8:58581166-58601463	TNUM	QTL:4253	Teat number	Chr.8:38873367-90653103	
		**TNB**	QTL:24282	Total number born	Chr.8:3470575-143577862	LW, ME
		**OVRATE**	QTL:492	Corpus luteum number	Chr.8:52718097-133922781	
OVOL1	2:5630670-5642984	**21DWT**	QTL:928	Body weight (3 weeks)	Chr.2:2387169-13366532	
PDK4	9:82625076-82638263	**OVRATE**	QTL:517	Corpus luteum number	Chr.9:45173556-138764263	
PION	9:113183480-113208758	**OVRATE**	QTL:517	Corpus luteum number	Chr.9:45173556-138764263	
PTHLH	5:49160138-49172386	**NSB**	QTL:18128	Number of stillborn	Chr.5:231749-108789684	LW, LD
SCNN1G	3:23444853-23480156	**OVRATE**	QTL:4249	Corpus luteum number	Chr.3:14776389-23924094	
		TNUM	QTL:5224	Teat number	Chr.3:1802851-131434519	LW, ME

		**BW**	QTL:5694	Body weight (birth)	Chr.3:14776389-62780348	DU, PT
		**DRIPL**	QTL:5692	Drip loss	Chr.3:14776389-62780348	DU, PT
SDCBP2	17:38419446-38437273	WWT	QTL:5231	Body weight (weaning)	Chr.17:3115596-69701581	LW, ME
		TNUM	QTL:5229	Teat number	Chr.17:13961137-69701581	LW, ME
SGPP2	15:137861023-137905123	TNB	QTL:22919	Total number born	Chr.15:134994861-138620895	DU, YS, LD
		**NBA**	QTL:22930	Total number born alive	Chr.15:134994861-138620895	DU, YS, LD
SH3BGR	13:213286139-213348206	**Wt**	QTL:1139	Body weight (5 weeks)	Chr.13:206615577-218635234	
TM9SF2	11:75703401-75712182	**NSB**	QTL:7534	Number of stillborn	Chr.11:52388584-78227264	French LD, LW
un-annotated	13:3656448-3695027	**NSB**	QTL:18133	Number of stillborn	Chr.13:3477201-3702865	LW, LD
un-annotated	15:117232153-117235744	**OVRATE**	QTL:10614	Corpus luteum number	Chr.15:114074540-153054254	ME, YS
un-annotated	15:122380337-122380596	**OVRATE**	QTL:10614	Corpus luteum number	Chr.15:114074540-153054254	ME, YS
un-annotated	3:100325999-100326309	TNUM	QTL:5224	Teat number	Chr.3:1802851-131434519	LW, ME
		**BW**	QTL:5234	Body weight (end of test)	Chr.3:2742110-138643006	LW, ME
un-annotated	6:51851923-51854793	**TNB**	QTL:24281	Total number born	Chr.6:8144130-116389124	LW, ME
un-annotated	3:10903133-10908516	TNUM	QTL:5224	Teat number	Chr.3:1802851-131434519	LW, ME
		**OVRATE**	QTL:515	Corpus luteum number	Chr.3:2847860-90815870	
un-annotated	6:54625398-54625615	**TNB**	QTL:24281	Total number born	Chr.6:8144130-116389124	LW, ME

Table S4. Differential expressed miRNAs found in small RNA libraries between both extreme phenotypes. All expression values are shown as RPKM values (Reads per Kilobase of exon model per Million mapped reads – Mortazavi et al., 2008). Mean difference between both groups is represented as the $\log_2$ transformed fold change (Log_2FC). Shown *q-values* are Benjamini-Hochberg FDR corrected *p-values.*

miRNA	miRNA precursor	High (RPKM)	Low (RPKM)	Log_2 FC	*p-value*	*q-value*
ssc-let-7c	*ssc-let-7c*	9,495.15	18,854.07	0.99	0.029	0.985
ssc-miR-31	*ssc-mir-31*	56.93	7.46	-2.933	0.004	0.985
ssc-miR-92a	*ssc-mir-92a-2*	51,874.13	21,710.41	-1.257	0.032	0.985
ssc-miR-92a	*ssc-mir-92a-1*	56,610.92	23,545.2	-1.266	0.035	0.985
ssc-miR-101	*ssc-mir-101-1*	430.21	187.19	-1.2	0.034	0.985
ssc-miR-101	*ssc-mir-101-2*	414.3	181.3	-1.192	0.038	0.985
ssc-miR-129a	*ssc-mir-129a*	4,616.22	17,489.47	1.922	0.047	0.985
ssc-miR-144	*ssc-mir-144*	10.64	0.76	-3.805	0.011	0.985
ssc-miR-145-5p	*ssc-mir-145*	34,958.9	81,171.84	1.215	0.045	0.985
ssc-miR-181d-5p	*ssc-mir-181d*	55.51	124.35	1.164	0.046	0.985
ssc-miR-382	*ssc-mir-382*	15.82	45.28	1.517	0.019	0.985
ssc-miR-450c-5p	*ssc-mir-450c*	170.41	411.28	1.271	0.017	0.985

Table S5. TargetScan results showing DEG predicted as target mRNAs for our differentially expressed miRNAs. [a] Sum of the contribution of site-type, 3' pairing, local AU, position, TA (target site abundance) and SPS (seed-pairing stability) calculated as in Garcia et al., 2011 [44].

[b] Probability of conserved targeting as described in Friedman et al., 2009 [45]

Representative miRNA	Representative Transcript	Target gene	Conserved sites				Poorly conserved sites				Total context +score[a]	Aggregate P_{CT}[b]
			Total	8mer	7mer-m8	7mer-1A	total	8mer	7mer-m8	7mer-1A		
hsa-miR-101	NM_053044	*HTRA3*	2	1	0	1	0	0	0	0	-0.49	0.94
	NM_001677	*ATP1B1*	2	0	0	2	0	0	0	0	-0.24	0.52
	NM_000963	*PTGS2*	1	1	0	0	0	0	0	0	-0.22	0.80
	NM_002229	*JUNB*	1	0	1	0	0	0	0	0	-0.06	0.42
hsa-miR-133a	NM_001977	*ENPEP*	1	0	1	0	1	0	0	1	-0.41	0.80
hsa-miR-144	NM_198965	*PTHLH*	1	1	0	0	0	0	0	0	-0.33	< 0.1
	NM_024420	*PLA2G4A*	1	1	0	0	0	0	0	0	-0.30	< 0.1
	NM_001677	*ATP1B1*	2	0	0	2	0	0	0	0	-0.22	0.52
	NM_053044	*HTRA3*	2	0	0	2	0	0	0	0	-0.21	0.52
	NM_000963	*PTGS2*	1	0	0	1	0	0	0	0	-0.06	0.31
hsa-miR-145	NM_001730	*KLF5*	1	1	0	0	0	0	0	0	-0.41	0.47
	NM_021154	*PSAT1*	1	1	0	0	0	0	0	0	-0.30	0.30
	NM_207446	*FAM174B*	1	1	0	0	0	0	0	0	-0.23	0.21
	NM_024420	*PLA2G4A*	1	0	1	0	1	0	0	1	-0.20	0.37
	NM_021914	*CFL2*	1	0	1	0	0	0	0	0	-0.09	0.50
hsa-miR-181d	NM_000902	*MME*	1	0	1	0	1	1	0	0	-0.17	0.35

Table S6. Candidate novel miRNAs predicted by miRDeep in H and L small RNA libraries. Shown RPKM values represent all read counts mapping in each novel miRNA mature, loop and/or star sequence. [a] A miRDeep score of 10 corresponds to a probability of> 90% to be a true positive.

Samples	Coordinates novel miRNA	miRDeep score[a]	Estimated Prob. of True Positive	Total RPKM	Mature RPKM	Loop RPKM	Star RPKM	Significant *p-value*	miRBase miRNA
High	chr5:1-111506441_34692	2.7e+5	0.95 ± 0.04	542,935	542,903	1	31	Yes	-
	chr6:1-157765593_36025	1.9e+5	0.95 ± 0.04	382,423	382,348	0	75	Yes	-
	chr13:1-218635234_6943	2.2e+4	0.95 ± 0.04	43,754	43,643	0	111	Yes	-
	chr5:1-111506441_18208	7.4e+5	0.93 ± 0.06	1,452,784	1,452,741	4	39	Yes	-
	chr6:1-157765593_19031	3.3e+5	0.93 ± 0.06	652,737	652,542	0	195	Yes	-
	chr13:1-218635234_3741	1.7e+4	0.93 ± 0.06	33,759	33,661	0	98	Yes	-
	chr5:1-111506441_23908	9.8e+5	0.94 ± 0.07	1,927,410	1,927,367	0	43	Yes	-
	chr6:1-157765593_24995	6.7e+5	0.94 ± 0.07	1,324,843	1,324,625	0	218	Yes	-
	chr13:1-218635234_4737	1.2e+4	0.94 ± 0.07	24,740	24,628	0	112	Yes	-
	chr6:1-157765593_31265	1.7e+5	0.96 ± 0.04	336,555	336,469	0	86	Yes	-
	chr5:1-111506441_30154	1.7e+5	0.96 ± 0.04	336,539	336,517	0	22	Yes	-
	chr13:1-218635234_5831	1.3e+4	0.96 ± 0.04	27,002	26,627	0	375	Yes	-
	chr5:1-111506441_7732	4.3e+5	0.92 ± 0.06	862,243	862,225	0	18	Yes	-
	chr6:1-157765593_8073	3.8e+5	0.92 ± 0.06	748,946	748,675	0	271	Yes	-
	chr13:1-218635234_1581	1.3e+4	0.92 ± 0.06	25,872	25,834	0	38	Yes	-
Low	chr6:1-157765593_18277	1.1e+5	0.93 ± 0.05	230,090	230,073	0	17	Yes	-
	chr5:1-111506441_17574	9.8e+4	0.93 ± 0.05	194,035	194,021	0	14	Yes	-
	chr13:1-218635234_3401	1.4e+4	0.93 ± 0.05	28,734	28,476	0	258	Yes	-
	chr5:1-111506441_34762	4.2e+5	0.95 ± 0.05	832,924	832,866	0	58	Yes	-
	chr6:1-157765593_36167	3.7e+5	0.95 ± 0.05	736,278	736,125	0	153	Yes	-
	chr13:1-218635234_6891	3.3e+4	0.95 ± 0.05	65,306	65,166	0	140	Yes	-
	chr5:1-111506441_18102	2.1e+5	0.92 ± 0.06	424,999	424,985	0	14	Yes	-
	chr6:1-157765593_18897	1.3e+5	0.92 ± 0.06	270,022	269,967	0	55	Yes	-

chr13:1-218635234_3579	1.7e+4	0.92 ± 0.06	35,288	35,200	0	88	Yes	-
chr5:1-111506441_13996	4.0e+5	0.95 ± 0.05	785,564	785,561	0	3	Yes	-
chr6:1-157765593_14561	3.1e+5	0.95 ± 0.05	621,594	621,384	0	210	Yes	-
chr13:1-218635234_2831	1.4e+4	0.95 ± 0.05	27,973	27,910	0	63	Yes	-
